# *Tis21* is required for adult neurogenesis in the subventricular zone and for olfactory behavior regulating cyclins, *BMP4*, *Hes1/5* and *Id*s

**DOI:** 10.3389/fncel.2014.00098

**Published:** 2014-04-07

**Authors:** Stefano Farioli-Vecchioli, Manuela Ceccarelli, Daniele Saraulli, Laura Micheli, Sara Cannas, Francesca D’Alessandro, Raffaella Scardigli, Luca Leonardi, Irene Cinà, Marco Costanzi, Andrea Mattera, Vincenzo Cestari, Felice Tirone

**Affiliations:** ^1^Institute of Cell Biology and Neurobiology, National Research Council, Fondazione Santa LuciaRome, Italy; ^2^Department of Psychology and “Daniel Bovet” Center, Sapienza University of RomeRome, Italy; ^3^Institute of Translational Pharmacology, National Research Council, Fondazione EBRIRome, Italy; ^4^Libera Università Maria Sartissima AssuntaRome, Italy

**Keywords:** adult neurogenesis, subventricular zone, *Hes1/5*, bone morphogenic proteins, *Id3*, stem cells, *Btg2*, olfactory memory

## Abstract

Bone morphogenic proteins (BMPs) and the *Notch* pathway regulate quiescence and self-renewal of stem cells of the subventricular zone (SVZ), an adult neurogenic niche. Here we analyze the role at the intersection of these pathways of *Tis21* (*Btg2/PC3*), a gene regulating proliferation and differentiation of adult SVZ stem and progenitor cells. In *Tis21*-null SVZ and cultured neurospheres, we observed a strong decrease in the expression of *BMP4 * and its effectors *Smad1/8*, while the *Notch* anti-neural mediators *Hes1/5* and the basic helix-loop-helix (bHLH) inhibitors *Id1-3* increased. Consistently, expression of the proneural bHLH gene *NeuroD1* decreased. Moreover, *cyclins D1/2*, *A2*, and *E* were strongly up-regulated. Thus, in the SVZ *Tis21* activates the BMP pathway and inhibits the *Notch* pathway and the cell cycle. Correspondingly, the *Tis21*-null SVZ stem cells greatly increased; nonetheless, the proliferating neuroblasts diminished, whereas the post-mitotic neuroblasts paradoxically accumulated in SVZ, failing to migrate along the rostral migratory stream to the olfactory bulb. The ability, however, of neuroblasts to migrate from SVZ explants was not affected, suggesting that *Tis21*-null neuroblasts do not migrate to the olfactory bulb because of a defect in terminal differentiation. Notably, BMP4 addition or *Id3* silencing rescued the defective differentiation observed in *Tis21*-null neurospheres, indicating that they mediate the *Tis21* pro-differentiative action. The reduced number of granule neurons in the *Tis21*-null olfactory bulb led to a defect in olfactory detection threshold, without effect on olfactory memory, also suggesting that within olfactory circuits new granule neurons play a primary role in odor sensitivity rather than in memory.

## INTRODUCTION

The subventricular zone (SVZ) of the lateral ventricles is one of the two neurogenic niches, together with the dentate gyrus of the hippocampus, where new neurons are continuously generated throughout adulthood (Zhao et al., 2008). According to a prevailing hypothesis, the new neurons in the SVZ are generated by resident radial glia-like cells that represent quiescent neural stem cells (reviewed by Alvarez-Buylla and Lim, 2004). These are a glial fibrillary acidic protein-positive (GFAP) subset of astrocytes, termed type B cells (Doetsch et al., 1999), which give rise to rapidly proliferating transient amplifying cells which are GFAP-negative and express the transcription factors of the Dlx family (type C cells; Doetsch et al., 2002). These type C cells in turn generate migrating neuroblasts, which are negative for GFAP but express Dlx2, PSA-NCAM (polysialylated neural adhesion cell molecule) and doublecortin (DCX; type A cells; Colak et al., 2008). Indeed, type B GFAP-positive neural stem cells in the SVZ are relatively quiescent, as they are less susceptible to antimitotic treatment (Doetsch et al., 1999).

Several morphogens and genes control the proliferation and maturation of stem cells and neuroblasts in the SVZ. These include *Notch*, *Sonic hedgehog* (*Shh*), and *bone morphogenic proteins* (*BMP*s). *BMP*s have been described to play a proastrocytic role by inhibiting neurogenesis when added to cultured SVZ neural stem cells and during embryonic development. Moreover, ependymal cells release the protein Noggin that may promote SVZ neurogenesis by antagonizing *BMP* signaling (Lim et al., 2000). However, recent work also indicates that the inhibition of *BMP* signaling – by ablating Smad4, an effector of *BMP4* – is required for the generation of type A neuroblast cells (Colak et al., 2008). A possibility which reconciles these views is that BMPs are required to maintain quiescence as well as self-renewal of SVZ stem cells, as observed in the dentate gyrus (Mira et al., 2010).

Furthermore, in the adult SVZ as well as in the dentate gyrus, the deletion of recombination signal-binding protein 1 (RBPJ), a downstream mediator of *Notch* receptors, triggers radial glia-like stem cells to differentiate into transient amplifying cells, causing the depletion of quiescent neural stem cells and the impairment of continuous neurogenesis (Ehm et al., 2010; Imayoshi et al., 2010). Proneural genes induce the ligand of *Notch*, i.e., Delta1, which activates *Notch* in neighboring cells, preventing their differentiation (Bray, 2006; Kageyama et al., 2009). Interestingly, the RBPJ-*Notch* pathway is linked to the cell cycle, as *cyclin D1* activates the transcription of *Notch* by recruiting CREB-binding protein (CBP) to its promoter, and *cyclin D1* and *Notch* exert the same effect of amplification of the progenitor population (Kageyama et al., 2009; Latasa et al., 2009; Bienvenu et al., 2010).

However, the interplay between *Delta1/Notch*, *BMP* and proneural genes implies other molecules deputed to trigger the exit from the cell cycle of the prospective neuron and to fine-tune the connection between cell cycle and proneural genes. An example could be the transcriptional cofactor *PC3/Tis21* (*Btg2*), whose expression is induced in the neuroblast at its last asymmetric mitosis (Iacopetti et al., 1994, 1999). *PC3/Tis21* induces the proliferating neural progenitor cells of the cerebellum, dentate gyrus and SVZ to exit the cell cycle and to differentiate by activating proneural genes through direct repression of the promoters of *cyclin D1* and of the inhibitor of proneural basic helix-loop-helix (bHLH) genes *Id3*, respectively (Canzoniere et al., 2004; Farioli-Vecchioli et al., 2007, 2008, 2009; Tirone et al., 2013). These two actions are distinct and require *PC3/Tis21*, as is evident in hippocampal dentate gyrus progenitor cells, where ablation of *PC3/Tis21* not only accelerates their proliferation, but also impairs terminal differentiation of early post-mitotic dentate gyrus neurons, although they have already exited the cell cycle (Farioli-Vecchioli et al., 2009). Moreover, ablation of *PC3/Tis21* in the SVZ has been shown to cause an increase of proliferation of stem/progenitor cells, consistently with its antiproliferative activity, and a decrease of SVZ neurons migrating to the olfactory bulb, their final migratory destination (Farioli-Vecchioli et al., 2009). As *PC3/Tis21* is activated by Delta1 and binds *in vitro* the BMP mediators *Smad1* and *Smad8* (Park et al., 2004; Hämmerle and Tejedor, 2007), we sought to further investigate in the SVZ how *PC3/Tis21* regulates the amplification and differentiation of progenitor cells and how it interacts with the main SVZ pathways.

We found that the ablation of *PC3/Tis21* (hereafter referred to simply as* Tis21*) impairs the expression of *BMP4* and of its effectors *Smad1/8*, whereas the mediators of the *Notch* pathway, *Hes1* and *Hes5*, increase. In addition, in the absence of *Tis21*, cyclins are highly induced. In cellular terms, this is associated to a large increase of self-renewal of stem cells, accompanied, however, by a defect of terminal differentiation of neuroblasts in the SVZ as well as in cultured neurospheres. This latter defect is rescued by BMP4 treatment or *Id3* silencing, revealing the role of these molecules in the *Tis21*-dependent differentiation. The defective differentiation of SVZ neuroblasts appears to be at the origin of their reduced migration to the outer region of the olfactory bulb. Such a defect causes a reduced sensitivity to odors, highlighting the importance of the contribution of granule cells (GCs) in local mitral/GC circuits.

Noteworthy, in damaged brains, for instance after ischemic stroke, the SVZ stem/progenitor cells may produce new neurons which are redirected toward the damaged area (Christie and Turnley, 2013). Our data suggest that Tis21, or BMP4 and Id3 are potential targets that can be manipulated in order to control neurogenesis after neural damage.

## MATERIALS AND METHODS

### MOUSE LINES AND GENOTYPING

The *Tis21* knockout mice had been generated previously, as described (Park et al., 2004). Mutant mice were of the C57BL/6 (B6) strain and had a replacement of the entire exon II of the *Tis21* gene. Genotyping of mice was routinely performed by polymerase chain reaction (PCR), using genomic DNA from tail tips, as described (Farioli-Vecchioli et al., 2009). Mice were maintained under standard specific-pathogen-free conditions, and all animal procedures were completed in accordance with the Istituto Superiore di Sanita’ (Italian Ministry of Health) and current European (directive 2010/63/EU) Ethical Committee guidelines.

### *IN SITU* HYBRIDIZATION

Preparation of sections (20 μm) and hybridization were performed as reported previously (Canzoniere et al., 2004). Antisense probes detecting mouse *Id3*, *Hes5*, *Mash1*, or *cyclin D1* mRNAs were synthesized by SP6 (or T7 for *cyclin D1*) polymerase from the pcDNA3 vector, in whose HindIII 5′-EcoRI 3′ sites (or BamHI 5′-EcoRI 3′ sites for *cyclin D1*) we cloned a specific, non-cross-reactive region of the cDNA. The antisense probes for *BMP4* and *Tis21* were synthesized by T7 polymerase from the PBR2.1 or from the pEX-A vectors, respectively, in whose KpnI 5′-XbaI 3′ or XbaI 5′-NotI 3′ sites we cloned the *BMP4*- or the *Tis21*-specific cDNA regions. The non-cross-reactive mRNA region was identified for each gene using the software Beacon Designer (Premier Biosoft, Palo Alto, CA, USA). As a template for amplification we used either an IMAGE clone or genomic mouse DNA. All clones were checked by sequencing. Riboprobes were labeled with digoxigenin-UTP (Transcription kit; Roche Products), following the protocol of the manufacturer. No signal was detected by the sense probe.

### BrdU TREATMENT OF MICE AND SAMPLE PREPARATION FOR IMMUNOHISTOCHEMISTRY

To detect SVZ stem and neuroblast cells entering the S phase, P60 mice were perfused 2 h after treatment with a single injection of Bromodeoxyuridine (BrdU; 95 mg/kg i.p.; **Figure 2**), according to previous protocols (Palma et al., 2005). To label slow-dividing stem cells, BrdU was given in the drinking water (1 mg/ml) of P46 mice for 2 weeks followed by 2 weeks of BrdU-free water, as described (Colak et al., 2008; **Figure 4**). SVZ neurons migrating in the rostral migratory stream (RMS) were detected by treating P60 mice with 5 daily injections of BrdU (95 mg/kg i.p.), followed by perfusion 6 days after the last injection (P71; **Figure 5**), according to a described protocol that allows detection of migrating neurons (Wittko et al., 2009; **Figure 5**). Moreover, 28-day-old neurons present in the olfactory bulb after migration from the SVZ were detected after treatment with five daily injections of BrdU from P60 to P64 (95 mg/kg i.p.), followed by perfusion at P88 (**Figure 6**).

Brains were collected after transcardiac perfusion with 4% paraformaldehyde (PFA) in phosphate buffered saline-diethyl pyrocarbonate (PBS-DEPC) and kept overnight in PFA. Afterward, brains were equilibrated in sucrose 30% and cryopreserved at -80°C.

### IMMUNOHISTOCHEMISTRY

Immunohistochemistry on SVZ, RMS, and olfactory bulb was performed on serial freefloating sections cut at 40 μm thickness using a cryostat at -25°C, from brains embedded in Tissue-Tek OCT (Sakura, Torrence, CA, USA). Sections were then stained for multiple labeling using fluorescent methods. The incorporation of BrdU was detected after pretreatment of sections to denature the DNA with 2N HCl 45 min at 37°C and then with 0.1 M sodium borate buffer pH 8.5 for 10 min. Primary antibodies used were a rat monoclonal antibody against BrdU (AbDSerotech, Raleigh, NC, USA; MCA2060; 1:400), mouse monoclonal antibodies against PSA-NCAM (Millipore Bioscience, Temecula, CA, USA; MAB5324; 1:300) or Olig2 (Millipore Bioscience; Temecula, CA, USA; MABN50; 1:200), a rabbit monoclonal antibody against Ki67 (LabVision Corporation, Fremont, CA, USA; SP6; 1:200) or rabbit polyclonal antibodies against cleaved (activated) Caspase-3 (Cell Signaling Technology, Danvers, MA, USA; 9661; 1:100), GFAP (DakoCytomation, Denmark; Z0334; 1:250) or Calretinin (Swant, Bellinzona, Switzerland; 7699/4; 1:200), or goat polyclonal antibodies raised against GFAP (Santa Cruz Biotechnology, Santa Cruz, CA, USA; Sc-6170, 1:300) or DCX (Santa Cruz Biotechnology; Sc-8066, 1:300).

Secondary antibodies used to visualize the antigen were all from Jackson ImmunoResearch (West Grove, PA, USA), as follows: a donkey anti-rat monoclonal antiserum conjugated to Cy3 or TRITC (tetramethylrhodamine isothiocyanate; BrdU); a donkey anti-mouse antiserum conjugated to Alexa 647 or 488 (PSA-NCAM, Olig2); a donkey anti-rabbit antiserum conjugated to Alexa 488 or 647 or to Cy3 (Calretinin, GFAP, Ki67, Caspase-3), or a donkey anti-goat antiserum conjugated to Alexa 488 or TRITC (DCX, GFAP). Images of the immunostained sections were obtained by laser scanning confocal microscopy using a TCS SP5 microscope (Leica Microsystem). Analyses were performed in sequential scanning mode to rule out cross-bleeding between channels.

### QUANTIFICATION OF CELL NUMBERS AND VOLUMES

Cell numbers in the SVZ, RMS, and in the olfactory bulb were obtained by counting cells expressing specific markers, visualized with confocal microscopy throughout the whole rostrocaudal extent of these structures in one-in-six series of 40-μm freefloating coronal sections (240 μm apart). Cell numbers obtained for each SVZ, RMS, and olfactory bulb section were divided for the corresponding area of the section, as described (Colak et al., 2008; Farioli-Vecchioli et al., 2012a), in order to obtain the average number of SVZ, RMS, or olfactory bulb cells per square millimeter. Areas were obtained by tracing the outline of the whole SVZ, RMS, or olfactory bulb, identified by the presence of cell nuclei stained by Hoechst 33258 on a digital picture captured and measured using the I.A.S. software (Delta Sistemi, Rome, Italy). Three animals per group were analyzed. The I.A.S. software was also used to count labeled cells. The volumes of the olfactory bulbs were calculated as described (Petreanu and Alvarez-Buylla, 2002), multiplying the average olfactory bulb area by section thickness and by number of sections (one-in-six series of 40-μm coronal sections).

### SVZ NEURAL STEM CELLS ISOLATION AND ANALYSIS

Neurospheres were generated from wild-type or *Tis21-*null mice as previously described (Gritti et al., 2001; Farioli-Vecchioli et al., 2012a). Briefly, the entire SVZ region of 2-month-old mice was dissected from sagittally cut brains, incubated with digestion enzymes (1.33 mg/ml trypsin, 0.7 mg/ml hyaluronidase, and 0.2 mg/ml kynurenic acid) for 30 min at 37°C and then mechanically dissociated with small-bore Pasteur pipette. The single cells obtained were cultured in Dulbecco modified eagle medium F12 (DMEM/F12) supplemented with B27 and EGF (20 ng/ml) and bFGF (10 ng/ml) in a humidified incubator at 37°C in 5% CO2. Neurospheres were passaged every 4th day by mechanically dissociation into single cells.

For the differentiation assay, 100000 cells from wild-type or *Tis21* knockout neurospheres at passage five were seeded on matrigel-coated coverslips in 24-well plates and transfected with either pSR-neo-GFP-sh*Id3* or pSR-neo-GFP-sh*LUC* (see below), or treated with BMP4 (Abnova, Taipei, Taiwan); 36 h after transfection, or at the same time of BMP4 treatment, the cell cultures were induced to differentiate in differentiation medium (DMEM/F12 supplemented with B27 w/o growth factors). After 48 or 72 h cells were fixed in 4% PFA for 10 min at RT, permeabilized in 0.1% Triton X-100 in PBS and then incubated with the primary goat polyclonal antibody against DCX (SantaCruz Biotechnology; Sc-8066 1:300) or the mouse monoclonal antibody against β-Tubulin (TuJ1; Covance, Princeton, NJ, USA; MMS-435P; 1:250). Secondary antibodies used to visualize the antigen were either a donkey anti-goat antiserum conjugated to Cy2 or Cy3 and a donkey anti-mouse antiserum conjugated either to TRITC (Jackson ImmunoResearch). Nuclei were stained by Hoechst 33258. Images of the immunostained cells were obtained by an Olympus Optical (Tokyo, Japan) BX53 fluorescence microscope connected to a Spot RT3 camera (Diagnostic Instruments Inc, Sterling Heights, MI, USA). DCX- and Tubulin-positive cells were counted as a percentage of Hoechst positive-nuclei, or – in the analyses of shId3 effects – as a percentage of green fluorescent protein-positive (GFP-positive) cells. The GFP generated by the pSR-neo-GFP constructs was directly visualized by microscopy.

For mRNA expression analysis, total RNA from secondary neurospheres at passage 5 was extracted using Trizol reagent (Invitrogen, San Diego, CA, USA) following the manufacturer’s instructions and reverse-transcribed as previously described (Guardavaccaro et al., 2000). The mRNA expression was analyzed by real-time RT-PCR amplification, using SYBR Green dye chemistry in duplicate samples and a 7900HT System (Applied Biosystems, Foster City, CA, USA). The mRNA relative expression values were obtained by the comparative cycle-threshold method (Livak and Schmittgen, 2001), by normalizing to TATA binding protein as endogenous control. Statistical analysis of mRNA expression values was performed by Student’s *t-*test on data normalized to the endogenous control but not relativized in fold expression of the calibrator sample. Specific, non-cross-reactive real-time RT-PCR primers were designed by the software Beacon Designer 8.02 (Premier Biosoft International) from published murine cDNA sequences; their sequences are available on request.

### RETROVIRUS PRODUCTION AND *IN VIVO* INFECTION

The retroviral vector pCAG-IRES-GFP, kindly provided by Dr. Chichung Lie (Institute of Developmental Genetics, Germany; Jessberger et al., 2008; Jagasia et al., 2009), was used to express the cDNA of *Tis21* (i.e., the murine sequence) only in dividing neural cells. The full open reading frame of *Tis21* cDNA was cloned in the sites SfiI-5′/PmeI-3′ of pCAG-IRES-GFP, obtaining pCAG-IRES-GFP-*Tis21*. The construct was checked by DNA sequencing. Retroviruses were propagated as previously described (Farioli-Vecchioli et al., 2008). The concentrated virus solution (10^8^ pfu/ml) was infused (1.5 μl at 0.32 μl/min) by stereotaxic surgery into the right and left SVZ of anesthetized P60 *Tis21*-null mice (anteroposterior = 0 mm from bregma; mediolateral = ±1 mm; dorsoventral = -1.8 mm). Infected, GFP-positive cells were counted throughout the whole rostrocaudal extent of the SVZ.

### SVZ EXPLANT CULTURES

Brains were dissected and placed in ice in Hank’s balanced salt solution (HBSS), as described (Shinohara et al., 2012). Afterward, 1 mm coronal slices were cut using a coronal brain matrix, between coordinates -0.5 and -2.5 mm anteroposterior from the bregma. The lateral ventricle walls were removed from the above sections and cut under the stereomicroscope in pieces of 50–200 μm of diameter, which were then resuspended in Neurobasal A medium (Invitrogen) mixed 1:3 with Matrigel (BD Biosciences). The tissue pieces embedded in Matrigel were then plated onto 24-well dishes (BD Biosciences, NJ, USA) on ice, and then left for 10 min at 37°C to allow polymerization. The explants thus obtained were cultured in Neurobasal A medium supplemented with B27, Glutamax and a penicillin-streptomycin mixture (Invitrogen) at 37°C in humidified air containing 5% CO2. At the end of the experiments the explants were fixed in 4% PFA for 20 min, washed in PBS, and analyzed with an inverted microscope (Leica DM IRB; Leica Microsystems, Wetzlar, Germany). The migration was quantified as described (Shinohara et al., 2012) by measuring the area of the bright cellular region around an explant, normalized to the explant perimeter.

### DESIGN OF siRNAs

The 19-nucleotide siRNA sequences specific to mouse *Id3* were designed with the on-line Design Tool software (MWG, Ebersberg, Germany). The two best candidate sequences were used to synthesize a pair of 64-mer oligonucleotides that were annealed and cloned in the BglII-5′ HindIII-3′ sites of the pSUPER.retro-neo-GFP retroviral expression vector, according to the manufacturer’s instructions (Oligoengine, Inc., Seattle). The siRNA *Id3*-203 sequence was as follows: 5′-TCCTGCAGCGTGTCATAGA-3′; the siRNA *Id3*-190 sequence was: 5′-CCACTGCTACTCGCGCCTG-3′. The control sequence from the luciferase gene was 5′-ACGGATTACCAGGGATTTC-3′ (see also Micheli et al., 2011). The presence of the correct sequence cloned in pSUPER.retro-neo-GFP was confirmed by sequencing.

### GENERATION OF shRNA RECOMBINANT RETROVIRUSES AND INFECTIONS

Retroviruses from the pSUPER. retro-neo-GFP-sh*Id3*-203, pSUPER.retro-neo-GFP-sh*Id3*-190 and pSUPER.retro-neo-shLUC constructs were generated by transfecting them into the packaging Phoenix helper cells using Lipofectamine (Life Technologies, Carlsbad, CA, USA) and used for infection of C2C12 cells following a described protocol (Micheli et al., 2011).

### IMMUNOBLOTS IN C2C12 CELLS

Western blot analysis of C2C12 myoblasts was performed as described (Micheli et al., 2011). Briefly, cells were lysed by sonication in buffer containing 50 mM Tris-HCl, pH 7.4, 150 mM NaCl, 1 mM EDTA, 0.2% Nonidet P-40, with protease inhibitors 1 mM Na_3_VO_4_, 10 mM 2-glycerophosphate, 10 mM NaF, 5 mM ATP, 5 mM MgCl_2_. Proteins were electrophoretically separated by SDS-PAGE and transferred to nitrocellulose filters. Immunoblots were performed hybridizing filters to a rabbit polyclonal antibody against Id3 (Santa Cruz Biotechnology; Sc-490, 1:300); detection of the second antibody (goat anti-rabbit horseradish peroxidase-conjugated antibody; Jackson ImmunoResearch) was performed by chemiluminescent assay.

### OLFACTORY TESTS

Behavioral experiments were performed on adult, two- to three-month-old, animals, housed in standard breeding cages (26 × 21 × 13 cm) and kept in a regular 12 h light/dark cycle, with illumination started at 7:00, at a constant temperature of 21°C. Access to water and food was provided *ad libitum*. The experiments were performed during the second half of the light period (between 2:00 and 5:00 p.m.), in soundproof rooms.

#### Olfactory detection threshold

The test was performed as previously described (Breton-Provencher et al., 2009), with minor modifications. In a cage identical to those used to house the animals, with the floor covered by a thin layer of wood chip bedding, two tissue culture dishes (35 mm × 10 mm), whose covers had been previously drilled with eight small holes each to allow the odors to spread, were placed in close proximity to two opposite sides of the cage. Each culture dish, properly sealed with a narrow strip of Parafilm M, contained a piece of filter paper (1.5 cm × 1.5 cm) soaked, in one case, with odorless mineral oil (25 μl), as a control, and, in the other, with butyl butyrate [or, as alternatives, octanal, (-) carvone, and (+) limonene] (25 μl), diluted in mineral oil at different concentrations (all from Sigma-Aldrich, St. Louis, MO, USA). During four successive sessions, lasting 3 min each and separated by 15 min intervals, each mouse was exposed to the culture dishes, one of which containing progressively increasing concentrations of the odorant (10^-^^7^, 10^-^^5^, 10^4^, and 10^-^^3^ %). Each session was video-recorded, and the time the animals spent sniffing at the culture dishes, defined as a nasal contact with the dish within a 0.5 cm distance, was subsequently measured by using EthoVision software (Noldus Information Technology, Wageningen, The Netherlands). For the statistical analysis, an ‘odor preference’ index was calculated, as the ratio between the time spent investigating the odor, and the total sniffing time (odor plus mineral oil), so that index values between 0.50 and 1.00 were indicative of preference for the odor, compared to mineral oil.

#### Olfactory discrimination

The test was performed as previously described (Gheusi et al., 2000), with minor modifications. In a cage identical to those used to house the animals, each mouse was familiarized with octanal [or (-) carvone], as the odor of habituation, during four successive trials, and then exposed to acetophenone [or (+) carvone], as the odor of dishabituation, on the final trial. Each trial lasted 3 min, and the trials were separated by 15 min intervals. Tissue culture dishes, containing filter paper soaked with the odorants, were prepared in the same manner as described for the *olfactory detection threshold* test. For all the odorants, the chosen dilution was 10^-^^3^ % in mineral oil. Each trial was video-recorded, and the time the animals spent sniffing at the culture dish, defined as a nasal contact with the dish within a 0.5 cm distance, was subsequently measured by using EthoVision software.

#### Olfactory associative memory

The test was performed as previously described (Schellinck et al., 2001), with minor modifications. Four days prior to training, the animals were placed on a food restriction schedule, and fed sufficiently to maintain 80–85% of their free feeding weight. During the training, they received four 10 min trials *per* day, in cages identical to their home cages. In two of the trials, a tissue culture dish, diffusing octanal [or (-) carvone], was hidden beneath the surface of the wood chip bedding, and paired with sugar reinforcement buried into the bedding; in the remaining two trails, an identical dish, diffusing acetophenone [or (+) carvone], was hidden without sugar. The culture dishes were prepared in the same manner as described for the *olfactory detection threshold* test; for all the odorants, the chosen dilution was 10^-^^3^ % in mineral oil; an amount of 50 μl of the odor solution was used to saturate the filter paper placed inside the dishes. The order of the trials was counterbalanced across training. The test was performed on day five, in a larger cage (20 × 70 × 20 cm) made of transparent Plexiglass and divided into three equal compartments, with openings (6 cm × 6 cm) leading to both the end compartments. Two culture dishes, diffusing octanal and acetophenone [or (-) carvone and (+) carvone], were placed in the end compartments of the cage, abundantly covered by the chip bedding, with no reinforcement. The test was video-recorded, and the time the animals spent digging the bedding in close proximity of each dish was measured, during a 4 min period, by an experimenter blind to their genotype. A brief habituation session, lasting 2 min, preceded the test, during which the animals were given the opportunity to explore the apparatus, with no odorant inside. After the habituation, the animals were returned to their home cages for approximately 10 min, prior to the start of the test.

## RESULTS

### EXPRESSION OF BMP, *NOTCH*, AND CELL CYCLE MOLECULES IN *Tis21* KNOCKOUT SVZ AND NEUROSPHERES

**FIGURE 1:**
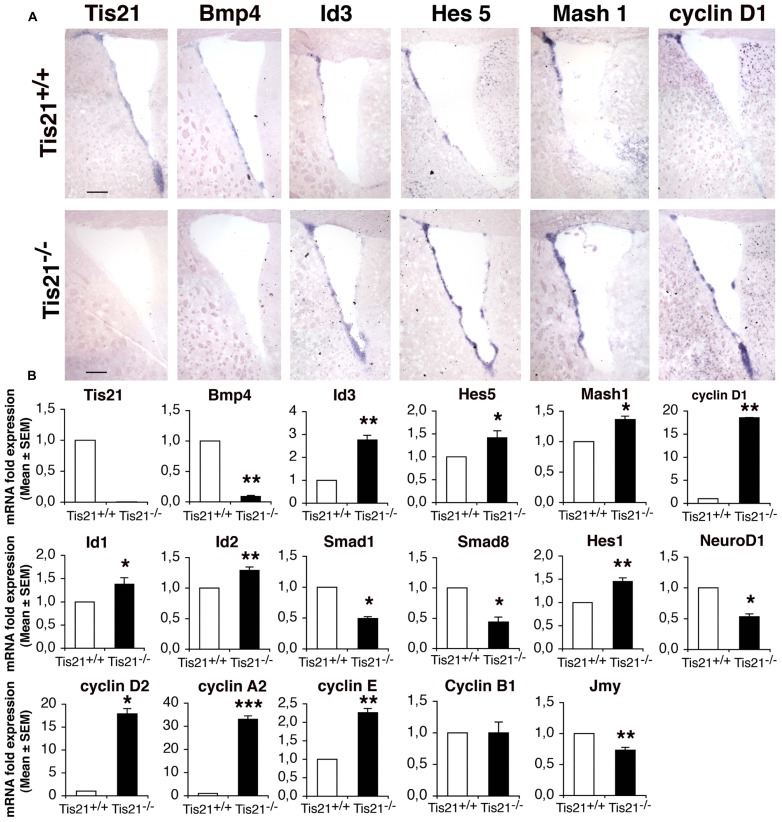
**Expression of the BMP, Notch, and cell cycle signaling components in the SVZ and in neurospheres of adult wild-type and *Tis21* knockout mice. (A)** *In situ* hybridization in coronal sections of SVZ from P60 *Tis21*-null and wild-type mice, of *Tis21, BMP4*, the downstream target Id3, the *Notch* effector *Hes5, the bHLH gene Mash1 and cyclin D1*. Scale bar 200 μm. (B) Real time PCR analysis of neurosphere cultures from SVZ neural stem cells of P60 mice; deprivation of *Tis21* results in the inhibition of *BMP4*, Smads and *NeuroD1* mRNAs, and increased expression of Ids, *Hes1/5* and cyclins mRNAs. Average ± SEM. values are from at least three independent experiments and are shown as fold change relative to the control sample (from wild-type mice), which was set to unit. TATA-binding protein mRNA was used as endogenous control for normalization. *p < 0.05, **p . 0.01, or ***p < 0.001 vs. *Tis21*+/+ SVZ; Student’s t–test.

Firstly, by *in situ* hybridization, we analyzed whether the ablation of *Tis21* affected expression of components of the BMP and of the *Notch* pathways, as well as of the cell cycle in the SVZ of adult (2-month-old) mice. A parallel analysis of mRNA expression was also performed on neurosphere cultures derived from the neural stem cells isolated from the SVZ of P60 mice, as previously described (Farioli-Vecchioli et al., 2012a). *Tis21* resulted clearly expressed in the dorsal and ventral SVZ (**Figures 1A,B**). The lack of *Tis21* led to a strong decrease of the expression of *BMP4* relative to the wild-type, in SVZ as well as in neurospheres (**Figures 1A,B**; *p* = 0.008). Consistently, the mRNAs of *Smad1* and *Smad8*, which bind *Tis21* and are activated by *BMP4* (Park et al., 2004; Miyazono et al., 2005), significantly decreased in *Tis21*-null neurospheres, as judged by real time PCR (**Figures 1A,B**; *p* = 0.033 and *p* = 0.023 for *Smad1* and *Smad8*, respectively). In contrast, the mRNA of the anti-differentiative *Id3* gene, which is activated by Smad proteins (Miyazawa et al., 2002; Shepherd et al., 2008) but is repressed by *Tis21* in dentate gyrus neurons (Farioli-Vecchioli et al., 2009), was significantly increased in *Tis21*-null SVZ with respect to the wild-type, as judged by *in situ* and real time PCR analyses (**Figures 1A,B**; *p* = 0.002). Similarly, Id1 and Id2 mRNAs were significantly increased in *Tis21*-null neurospheres, relative to the wild-type (**Figure 1B**; *p* = 0.03 for Id1 and *p* = 0.002 for Id2). As for the *Notch* pathway, we analyzed the bHLH inhibitory genes *Hes5* and *Hes1*, which, once activated in the nucleus by the intracellular domain of *Notch* complexed with RBPJ, repress the proneural bHLH genes, either by passively sequestering E 47, or by actively recruiting the co-repressor Groucho (Kageyama et al., 2008, 2007). *Hes5* and *Hes1* mRNAs resulted increased in *Tis21*-null SVZ and neurospheres, relative to the wild-type (**Figures 1A,B**; *p* = 0.044 for *Hes5* and *p* = 0.002 for *Hes1*). Furthermore, the mRNAs of *D cyclins* (*D1*, *D2*), *cyclin E* and *cyclin A2*, that regulate the entry and progression into S-phase, respectively (Johnson and Walker, 1999), were strongly induced in *Tis21*-null SVZ and neurospheres (**Figures 1A,B**; *p* = 0.01 for *cyclin D1*, p = 0.010 for *cyclin D2*, *p* = 0.0008 for *cyclin A2*, *p* = 0.007 for *cyclin E*). In the SVZ we also analyzed the expressions of the bHLH pro-neural genes *NeuroD1*, which is necessary for the differentiation of SVZ neurons (Gao et al., 2009), and *Mash1*, which is expressed in transient amplifying progenitor cells (type C) and is required for the generation of both neurons and oligodendrocytes (Parras et al., 2004). We observed that in *Tis21*-null neurospheres *NeuroD1* mRNA decreased relative to the wild-type, while *Mash1* mRNA increased both by *in situ* and by PCR analysis of neurospheres (**Figures 1A,B**; *p* = 0.018 for *NeuroD1*, *p* = 0.04 for *Mash1*). In SVZ neurospheres, we also analyzed the mRNA expression of genes controlling the migration of neurons and known to be regulated by *Tis21*, namely, the chemokines Cxcl3 and Cxcl12, Efna4, Pag1, and Jmy (Farioli-Vecchioli et al., 2012b). Of these genes, only Jmy is expressed in neurospheres, and this decreased significantly in the absence of *Tis21* (**Figure 1B**; *p* = 0.009).

**FIGURE 2:**
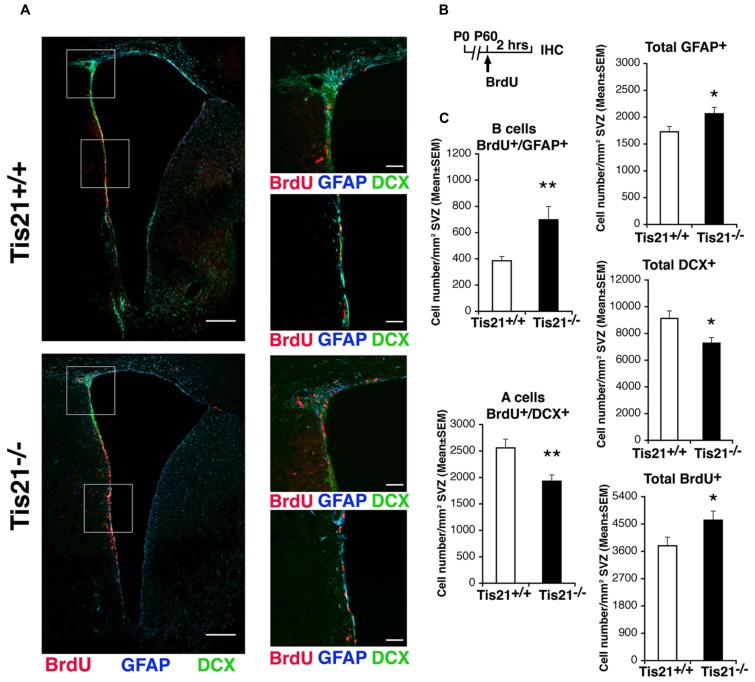
**Dividing stem cells increase while differentiating neuroblasts decrease in the SVZ of adult* Tis21* knockout mice. (A)** Representative confocal images of coronal sections of the SVZ in P60 *Tis21*^+^^/^^+^ and *Tis21*^-^^/^^-^ mice, showing dividing B stem cells, identified as double-labeled BrdU^+^/GFAP^+^ cells (red and blue, respectively), and A neuroblasts, identified as double-labeled BrdU^+^/DCX^+^ cells (red and green, respectively). On the right are higher magnification images of the dorsal and lateral SVZ areas indicated in white boxes. Scale bars, 200 μm and 50 μm (enlargement). **(B)** Scheme of treatment, with a single BrdU injection performed 2 h before analysis. **(C)** Quantification of the number per SVZ area in P60 mice of dividing B stem cells (BrdU^+^/GFAP^+^), of A neuroblast cells (BrdU^+^/DCX^+^) and total B (GFAP^+^) and A (DCX^+^) cells, as well as of total dividing cells (BrdU^+^). At P60 B cells increase strongly while A neuroblasts decrease, relative to the wild-type. Cell numbers are mean ± SEM of the analysis of three animals per group. *p < 0.05, or **p < 0.01 vs. *Tis21*^+^^/^^+^ SVZ; Student’s *t*-test.

We, therefore, analyzed the numbers of proliferating stem cells and neuroblasts in *Tis21*-null SVZ, as determined by incorporation of BrdU after a 2 h pulse. We observed that in P60 *Tis21*-null mice the proliferating type B astrocytic-like stem cells, identified as BrdU^+^GFAP^+^ cells, significantly increased relative to the wild-type, whereas the dividing type A neuroblasts, identified as BrdU^+^DCX^+^ cells (Zhao et al., 2008), significantly decreased (B cells, about 80% increase, *p* = 0.003; A cells, 25% decrease, *p* = 0.004; **Figures 2A–C**). Consistently, the total number of GFAP^+^ stem cells increased, whereas the total number of type A neuroblasts decreased (GFAP^+^: *p* = 0.040; DCX^+^: *p* = 0.012; **Figures 2A–C**). The total number of proliferating cells increased slightly but significantly (BrdU^+^: *p* = 0.046; **Figures 2A–C**). Thus, the whole population of type B stem cells increased, while type A neuroblasts cells appeared reduced in the SVZ of *Tis21*-null P60 mice.

We then tested the possibility that the decrease of A neuroblasts was a consequence of increased apoptosis, by activated Caspase-3 immunostaining (Samuel et al., 2007). However, we observed that no change occurred in the number of A neuroblasts undergoing apoptosis in *Tis21* knockout SVZ, relative to the wild-type (activated Caspase-3^+^/DCX^+^ cells/mm^2^ in *Tis21*^-^^/^^-^ SVZ: 55.8 ± 16.7 and in *Tis21*^+^^/^^+^ SVZ: 49.9 ± 18.6; *p* = 0.89, n [mice] = 3). It is known that inhibition of the BMP pathway in the SVZ (through ablation of Smad4) can induce a shift of the fate of neuroblasts toward oligodendrogliogenesis, by increasing the expression of Olig2 in a subset of transiently amplifying progenitors (type C cells; Colak et al., 2008). Thus, we verified whether the number of Olig2-positive cells changed in the SVZ; we observed no difference in the SVZ of *Tis21*-null mice, relative to the wild-type (Olig2^+^ cells/mm^2^ in *Tis21*^-^^/^^-^ SVZ: 2269 ± 104 and in *Tis21*^+^^/^^+^ SVZ: 2422 ± 96; *p* = 0.29, *n* [mice] = 3).

**FIGURE 3:**
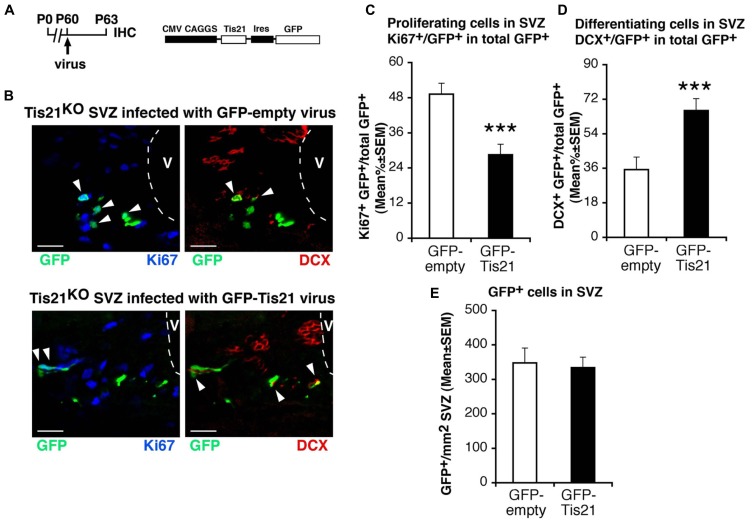
***Tis21*-retrovirus rescues the uninhibited proliferation and the defective differentiation of *Tis21*-null SVZ stem cells and neuroblasts. (A)** Scheme of retrovirus infection and analysis, and structure.** (B)** Representative confocal images of coronal sections in the dorsal SVZ region, double labeled with GFP (after infection with either GFP-*Tis21* or GFP-empty retroviruses for 72 h) and with Ki67 or DCX. The ventricle (V) is outlined by dashed lines. Scale bars, 25 μm. **(C)** Percentage ratio between Ki67^+^/GFP^+^ or **(D)** DCX^+^/GFP^+^ cells and the total number of infected cells (GFP^+^), from the analysis of *Tis21*^-^^/^^-^ SVZ infected with either GFP-*Tis21* or GFP-empty virus. The increase of dividing cells (Ki67^+^) and the decrease of differentiating neuroblasts (DCX^+^) is reversed by the *Tis21* virus, relative to the empty virus infections. **(E)** Total number per SVZ area of cells infected (GFP^+^) with either GFP-*Tis21* or GFP-empty virus. Cell numbers are mean ± SEM of the analysis of three animals per group. ***p < 0.001 vs. *Tis21*^-^^/^^-^ SVZ infected with GFP-empty virus; Student’s *t*-test.

Hence, the increase of the population of type B proliferating stem cells and the decrease of differentiating type A neuroblasts cells in *Tis21*-null SVZ is consistent with the known antiproliferative and pro-differentiative actions of *Tis21* (Tirone et al., 2013). By infecting the SVZ of *Tis21*-null mice with a retrovirus expressing *Tis21* and GFP (**Figure 3A**), the increase of dividing cells and the decrease of differentiating neuroblasts were completely reversed (Ki67^+^/GFP-*Tis21*^+^ vs. Ki67^+^/GFP-empty^+^ in *Tis21*-null mice *p* = 0.0001; **Figures 3B,C**; DCX^+^/GFP-*Tis21*^+^ vs. DCX^+^/GFP-empty^+^ in *Tis21*-null mice *p* = 0.0009; **Figures 3B,D**). No difference was observed in the number of cells per SVZ area infected by either the GFP-empty or GFP-*Tis21* retroviruses (**Figure 3E**). This indicates that the defect of differentiation of neuroblasts is reversible and specifically dependent on the loss of *Tis21*.

Moreover, when cells from secondary neurosphere cultures of *Tis21*-null SVZ were induced to differentiate, within 48 h they showed a decrease of the percentage of differentiating neurons relative to wild-type SVZ cultures (calculated as percentage ratio of DCX^+^ neuroblasts to the total number of cells, labeled by Hoechst 33258; *Tis21*^-^^/^^-^ DCX^+^/Hoechst^+^: 16.04 ± 1.1 %, *Tis21*^+^^/^^+^ DCX^+^/Hoechst^+^: 29.89 ± 2.1%, *p* < 0.00001, *n* [mice] = 3). This suggests that the defect of differentiation observed in SVZ neuroblasts of *Tis21*-null mice is cell-intrinsic.

**FIGURE 4:**
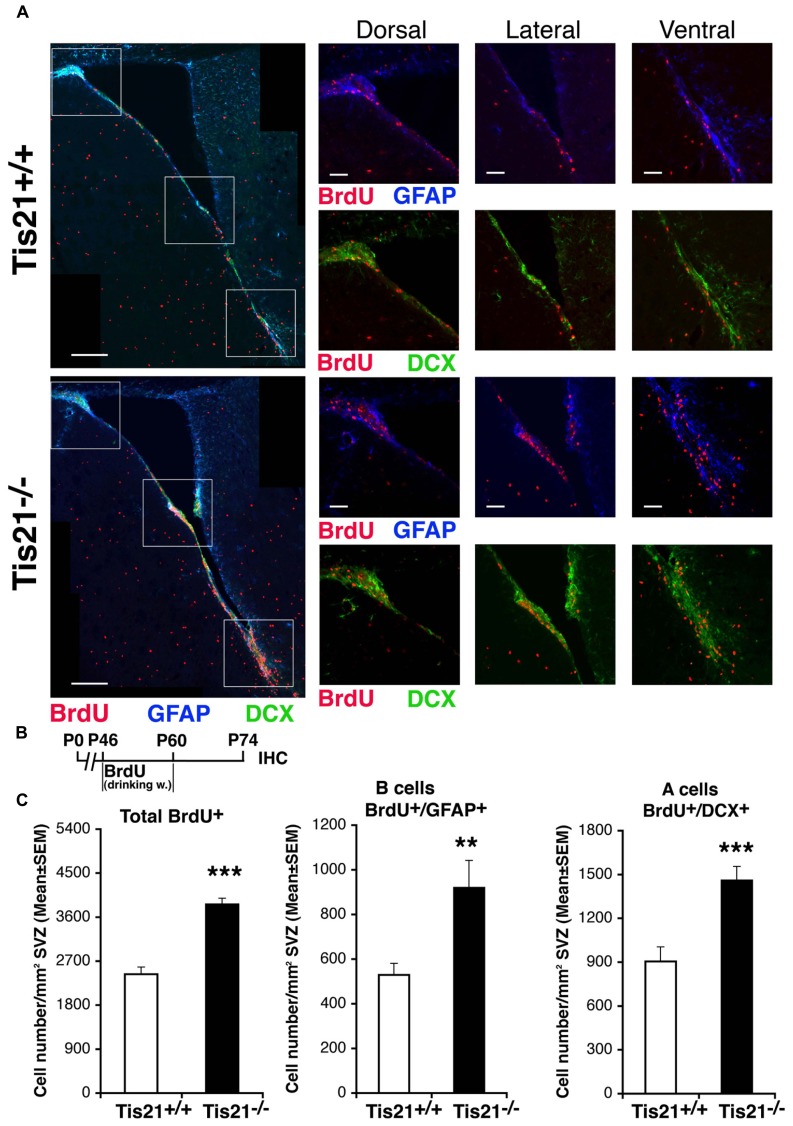
**Increase of slow-dividing stem cells and of neuroblasts that have exited the cell cycle in adult SVZ of *Tis21* knockout mice. (A)** Representative confocal images of coronal sections of the SVZ in P74 *Tis21*^+^^/^^+^ and *Tis21*^-^^/^^-^ mice, with higher magnification views of dorsal, lateral and ventral areas (white boxes). Shown are slowly dividing B stem cells (BrdU^+^/GFAP^+^) and BrdU-retaining A neuroblasts (BrdU^+^/DCX^+^). Scale bars, 200 and 50 μm (enlargement). **(B)** Scheme of BrdU treatment, added to drinking water for 2 weeks followed by 2 weeks without BrdU and analysis at P74. **(C)** Quantification per SVZ area in P74 mice of the number of the total (BrdU^+^) and B stem slow-dividing cells (BrdU^+^/GFAP^+^), as well as of BrdU-retaining A neuroblast cells (BrdU^+^/DCX^+^) that have exited cell cycle; all of them increase in number in *Tis21*^-^^/^^-^ mice relative to the wild-type. Cell numbers are mean ± SEM of the analysis of three animals per group. **p < 0.01, or ***p < 0.001, vs. *Tis21*^+^^/^^+^ SVZ; Student’s *t*-test.

We then sought to define *in vivo* the proportion of self-renewing stem cells affected by *Tis21* knockout, by means of a label-retaining protocol (Colak et al., 2008). The addition of BrdU to drinking water for 2 weeks followed by 2 weeks without BrdU allows labeling of cells that divide slowly, in contrast to cells that divide rapidly (such as transients amplifying cells type C) and thus dilute BrdU labeling. This protocol also labels neuroblasts (type A), which exit the cell cycle after incorporating BrdU. We observed that slow-dividing GFAP^+^ stem cells increased considerably in *Tis21*-null SVZ (57% increase, *p* = 0.005; **Figures 4A–C**), as well as BrdU-retaining neuroblasts (62% increase, *p* = 0.0004; **Figures 4A–C**). This indicated that the *Tis21* knockout-dependent increase of proliferation of stem cells concerned the self-renewing pool, in agreement with the data of **Figure 2**. It also indicated an unexpected accumulation in the SVZ of type A neuroblasts, which soon after differentiating should migrate to the olfactory bulb. This accumulation of BrdU-retaining *Tis21*-null neuroblasts suggested two possible causes, either a defect of terminal differentiation hindering their migration, or an intrinsic defect of migration. In fact, we could exclude a third possibility, i.e., an increased survival (see above).

**FIGURE 5:**
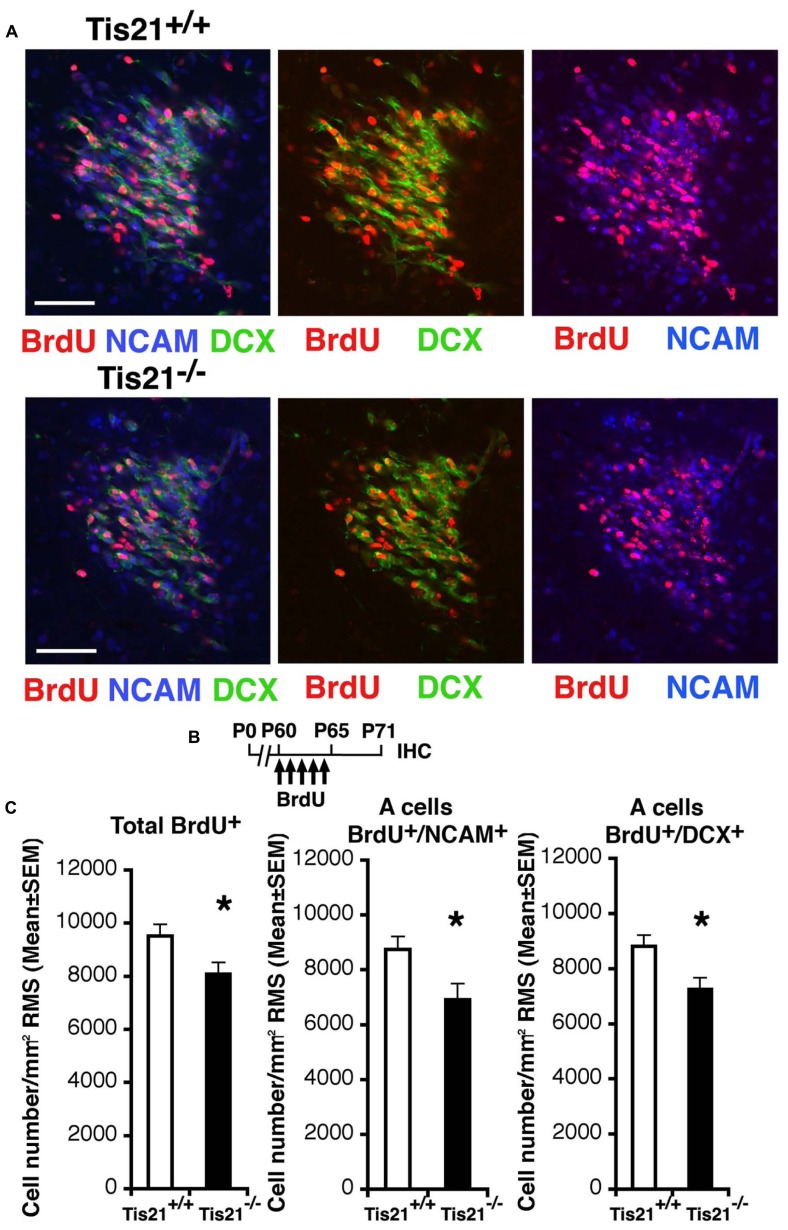
***Tis21* deletion results in decreased numbers of SVZ cells migrating through the RMS toward the olfactory bulb. (A)** Representative confocal images of coronal sections of the intermediate RMS in P71 *Tis21*^+^^/^^+^ and *Tis21*^-^^/^^-^ mice, labeled with three (on the left; BrdU^+^/NCAM^+^/DCX^+^) or two markers (center; BrdU^+^/DCX^+^; on the left; BrdU^+^/NCAM^+^). Scale bars, 50 μm. **(B)** Scheme of BrdU treatment, with five daily BrdU injection after P60 and analysis performed 11 days after (P71). **(C)** Quantification throughout the whole RMS in P71 mice of the number per RMS area of the total (BrdU^+^) cells and of the tangentially migrating A neuroblasts (BrdU^+^/NCAM^+^ or BrdU^+^/DCX^+^); the migrating neuroblasts decrease in number in *Tis21*^-^^/^^-^ mice relative to wild-type. Cell numbers are mean ± SEM of the analysis of three animals per group. *p < 0.05 vs. *Tis21*^+^^/^^+^ RMS; Student’s *t*-test.

Thus, we analyzed next the ability of neuroblasts to migrate to the olfactory bulb. In fact, the adult-generated neurons, once they are produced in the SVZ, migrate to the olfactory bulb through a complex path of migration, up to 5 mm long in rodents, called the RMS (Altman, 1969; Lois and Alvarez-Buylla, 1994). We analyzed the number of migrating neuroblasts in coronal sections along the whole RMS, identified through BrdU labeling as 11-day-old neurons, either BrdU^+^/DCX^+^ or BrdU^+^/PSA-NCAM^+^, since at this age new SVZ neuroblasts are in the process of tangentially migrating (Luskin, 1993; Wittko et al., 2009). PSA-NCAM is in fact required for the proper organization of the RMS and labels tangentially migrating type A neuroblasts (Doetsch et al., 1997; Lledo and Saghatelyan, 2005). We observed that in P60 *Tis21*-null mice the migrating neuroblasts, analyzed throughout the whole RMS from caudal to rostral (i.e., proximal to the SVZ or to the olfactory bulb, respectively), significantly decreased, relative to the wild-type (BrdU^+^/DCX^+^, *p* = 0.023; BrdU^+^/NCAM^+^, *p* = 0.012; **Figures 5A,B**). Moreover, this decrease of migrating neuroblasts did not depend on changes in RMS area or cell density, as these parameters did not show differences (average RMS area from coronal sections measured through the whole RMS: 19512 ± 1960 μm^2^ in wild-type and 22164 ± 2020 μm^2^ in *Tis21* knockout, *p* = 0.35; RMS cell density: 9822 ± 754 cells/mm^2^ in wild-type and 10982 ± 3116 cells/mm^2^ in *Tis21* knockout, *p* = 0.70; *n* [mice] = 3).

We further analyzed whether the decrease of migrating neuroblasts corresponded to a decrease of migrated neurons in the olfactory bulb, their final destination (Zhao et al., 2008). In the olfactory bulb, SVZ-derived neuroblasts generated postnatally differentiate into two types of local interneurons: GCs and periglomerular cells (Lois and Alvarez-Buylla, 1994). GCs are the predominant interneuron type generated in the adult and Calretinin turns out to be the most predominant marker for GCs generated during the postnatal period (Whitman and Greer, 2007; Batista-Brito et al., 2008).

**FIGURE 6:**
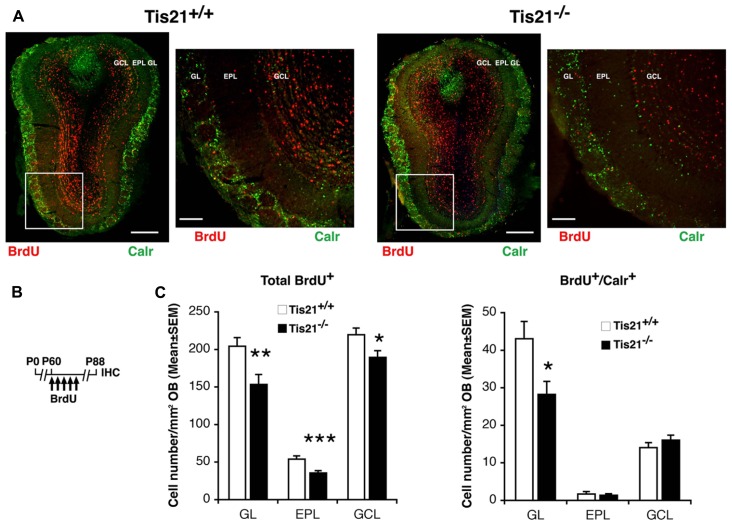
***Tis21* deletion results in decreased numbers of SVZ neuroblast-derived granule cells in the olfactory bulb. (A)** Representative confocal images (coronal sections) of the olfactory bulb in P88 *Tis21*^+^^/^^+^ and *Tis21*^-^^/^^-^ mice, showing terminally differentiated 28-day-old neurons, identified as total BrdU^+^ cells or as Calretinin^+^ neurons (red and green, respectively). On the right are higher magnification images of the regions in white boxes. Scale bars, 300 and 100 μm (enlargement). In the glomerular layer a decrease is evident of the BrdU^+^/Calretinin^+^ interneurons, derived from the SVZ neuroblasts that have migrated locally and terminally differentiated. **(B)** Scheme of BrdU treatment, with five daily BrdU injection after P60 and analysis performed 28 days after (P88). **(C)** Quantification in the three layers of olfactory bulb (OB) – the glomerular layer (GL), the intermediate external plexiform layer (EPL), and the internal granule cell layer (GCL) – of the number per area of the total 28-day-old differentiated neurons (BrdU^+^) and of the granule cell interneurons (BrdU^+^/Calretinin^+^); these latter decrease significantly in the glomerular layer of *Tis21*^-^^/^^-^ mice, the main region where granule cells interneurons differentiate and reside. Cell numbers are mean ± SEM of the analysis of three animals per group. *p < 0.05, **p < 0.01, or ***p < 0.001, vs. *Tis21*^+^^/^^+^ olfactory bulb; Student’s *t*-test.

Analyzing the number of 28-day-old Calretinin^+^ neurons in the olfactory bulb, we observed that in P60 *Tis21*-null mice they were significantly reduced, with respect to the wild-type, only in the glomerular layer (GL), while no evident change occurred in the intermediate external plexiform layer (EPL) and in the internal GC layer (GCL) (BrdU^+^/Calretinin^+^ in GL, *p* = 0.013; in EPL, *p* = 0.64; in GCL, *p* = 0.28; **Figures 6A,C**). This finding is consistent with the preferential presence of Calretinin^+^ GC neurons in the more external layer, i.e., the GL (Batista-Brito et al., 2008). In all three layers of the olfactory bulb we detected a significant decrease of 28-day-old neurons (BrdU^+^ in GL, *p* = 0.006; in EPL, *p* = 0.0009; in GCL, *p* = 0.021; **Figures 6A,C**) suggesting a general decrease in *Tis21*-null mice of the new neurons migrated from the SVZ to the olfactory bulb.

Furthermore, we tested whether the reduced number of new neurons in the olfactory bulb was related to non-specific changes, such as a reduced olfactory bulb volume. No significant difference was observed between *Tis21*-null and wild-type mice at P60 in the volumes of the olfactory bulb (5.93 ± 0.02 mm^3^ and 6.07 ± 0.49 mm^3^, respectively; *p* = 0.79, *n* = 3 [mice]).

**FIGURE 7:**
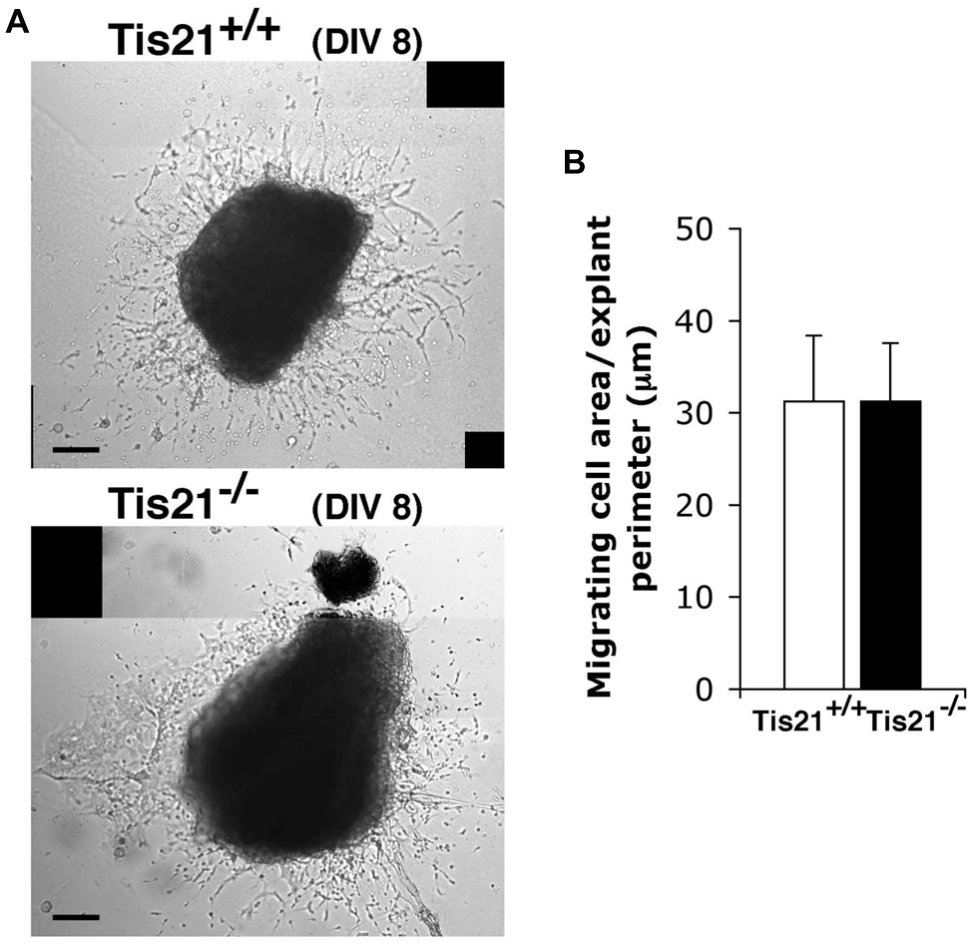
***Tis21* deletion does not affect the intrinsic migration of neurons from SVZ explants. (A)** Neuroblast migration from SVZ explants isolated from P60 *Tis21*-knockout and wild-type mice at 8 days (8 DIV) of the culture. The bright regions surrounding the explants in representative phase-contrast images are formed by cells that have migrated out. **(B)** Quantification of the migration of neuroblasts from SVZ explants (*n* = 3 mice for each genotype). The migration was quantified by measuring the area of the bright region around the explant, visible in **(A)**, after normalization to the perimeter of the explant. Scale bar 100 μm.

These data, however, did not clarify whether the decreased migration of *Tis21* knockout SVZ neuroblasts through the RMS to the olfactory bulb derived from an intrinsic defect of migration or was the consequence of an impairment of terminal differentiation (as suggested by the delayed differentiation observed *in vitro*, see above). To assess this point, we examined the migration of SVZ neuroblasts *in vitro*, by isolating explants of SVZ from P60 mice. These explants were cultured in Matrigel, in which SVZ neuroblasts migrate in chains (Wichterle et al., 1997; Shinohara et al., 2012). We did not detect any significant change in the ability of *Tis21*-null SVZ cells to migrate from the explant, relative to the wild-type (*p* = 0.9; **Figures 7A,B**). These findings suggest that *Tis21*-null SVZ neuroblasts are defective in terminal differentiation.

### THE DEFECT OF DIFFERENTIATION OF *Tis21*-null SVZ NEUROSPHERES IS RESCUED BY *Id3* SILENCING AND BY BMP4

We sought to analyze further the evidence of a defect in terminal differentiation of *Tis21*-null SVZ neuroblasts, by ascertaining the mechanism underlying the defective differentiation observed in *Tis21*-null SVZ neurospheres (see above). Specifically, we asked if this was dependent on the increase of *Id3* expression observed in *Tis21*-null SVZ, or even on the decrease of *BMP4*, whose pathway has been shown to control also the number of DCX^+^ neuroblasts (Colak et al., 2008).

**FIGURE 8:**
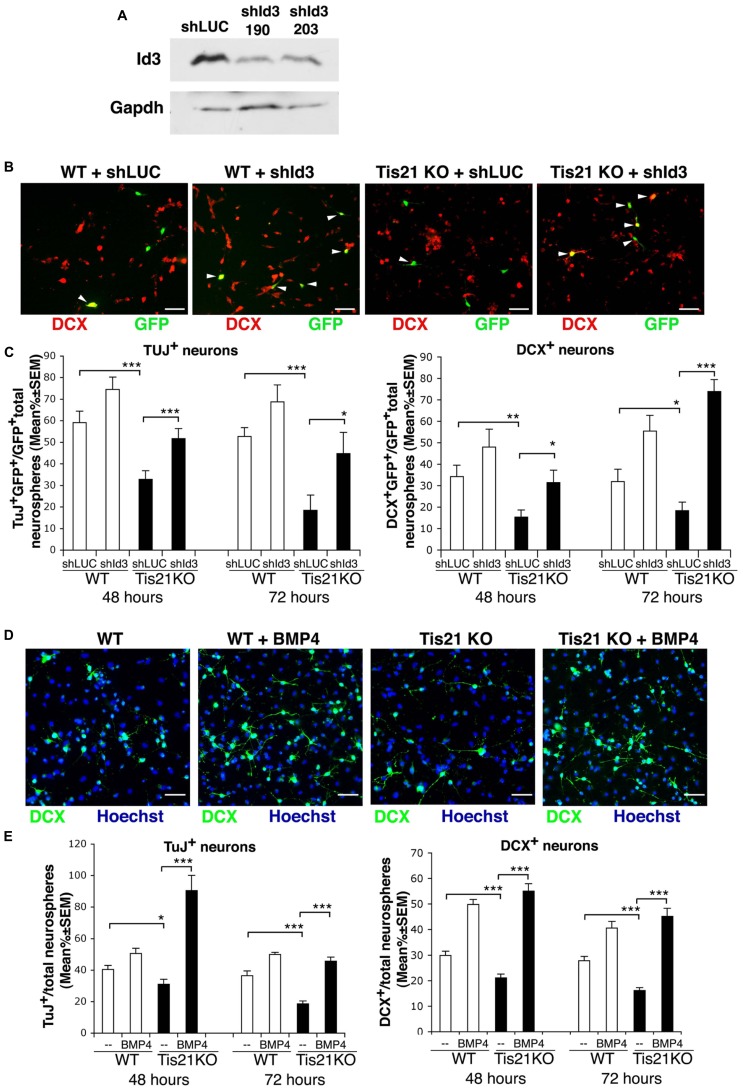
**Silencing of *Id3* expression or treatment with BMP4 in *Tis21*-null SVZ neurosphere cultures rescues their defect of differentiation. (A)** Analysis of Id3 protein expression in proliferating C2C12 myoblasts, infected with retroviruses generated by a pSUPER-retro vector expressing the *Id3*-specific shRNA sequences sh*Id3*-190 and sh*Id3*-203 or with a control retrovirus expressing an shRNA targeting luciferase (sh*LUC*). After infection, cells were selected for 10 days with G418 and then cultured in proliferating conditions. **(B)** Representative images of differentiated cells from secondary SVZ neurosphere cultures from 2-month-old wild-type or *Tis21* knockout mice, transfected 36 h prior to the onset of differentiation with either the pSR-neo-GFP-sh*Id3* or the pSR-neo-GFP-sh*LUC* constructs. The cells shown were analyzed 72 h after the start of differentiation; DCX^+^ cells were visualized in red, whereas cells transfected with success were green (GFP^+^). White arrowheads indicate double labeled cells (DCX^+^GFP^+^). Scale bar 50 μm. **(C)** Quantification of the percentage of early or late differentiated neurosphere-derived cells from *Tis21*-null and wild-type SVZ silenced for *Id3* or control, after transfection with the pSR-neo-GFP-sh*Id3* or the pSR-neo-GFP-sh*LUC* constructs, respectively. The percentage of early or late differentiated neurosphere cells was measured as the ratio between TuJ^+^GFP^+^ or DCX^+^GFP^+^ cells and total GFP^+^ cells, transfected with either pSR-neo-GFP-sh*Id3* or with pSR-neo-GFP-sh*LUC.* The analysis was performed 48 or 72 h after the shift to differentiation medium, as indicated. Mean percent values ± SEM are from three independent experiments. *p < 0.05, **p < 0.01, or ***p < 0.001; Student’s *t*-test. **(D)** Representative images of differentiated cells from secondary SVZ neurosphere cultures from 2-month-old wild-type or *Tis21* knockout mice, treated with either BMP4 (50 ng/ml) or control solution (vehicle). Cells were shifted to differentiation medium containing BMP4 and 72 h after the cells were analyzed; DCX^+^ cells were visualized in green. Scale bar 50 μm. **(E)** Quantification of the percentage of early or late differentiated neurosphere-derived cells from *Tis21*-null and wild-type SVZ treated with BMP4 (50 ng/ml) or with control solution. The percentage of early or late differentiated cells was measured as the ratio between TuJ^+^ or DCX^+^ cells and total cells (identified as Hoechst^+^). The analysis was performed 48 or 72 h after the shift to differentiation medium containing BMP4, as indicated. Mean percent values ± SEM are from three independent experiments. *p < 0.05, or ***p < 0.001; Student’s *t*-test.

First, we identified a retrovirally delivered shRNA specifically targeting *Id3*, by analyzing two candidate 19-nt *Id3* targeting sequences designed with the MWG on-line Design Tool software (MWG, Ebersberg, Germany). The sequences were cloned in the pSUPER.retro-neo-GFP vector (pSR-neo-GFP-sh*Id3*-190 and pSR-neo-GFP-sh*Id3*-203), and the corresponding retroviruses were generated. C2C12 myoblasts, a test line chosen for the high physiological level of *Id3* expression, were then infected with the retrovirus expressing the *Id3* targeting sequence or with a control retrovirus expressing an shRNA targeting luciferase (pSR-neo-GFP-sh*LUC;* Micheli et al., 2011; Farioli-Vecchioli et al., 2012b) and were selected for resistance to neomycin. The analysis of the derived myoblast populations showed that the candidate shRNA sequences were capable of silencing *Id3* protein expression (**Figure 8A**). The pSR-neo-GFP-sh*Id3*-190 (hereafter named pSR-neo-GFP-sh*Id3*) was selected for further experiments.

Thus, single cells from secondary neurosphere cultures of *Tis21*-null SVZ from 2-month-old mice were transfected with either pSR-neo-GFP-sh*Id3*, the retroviral vector expressing GFP and the shRNA targeting *Id3*, or with the control pSR-neo-GFP-sh*LUC*; alternatively cell cultures were treated with BMP4 or with control solution. 36 h after transfection, or at the same time as BMP4 treatment, the cell cultures were induced to differentiate in differentiation medium for 48 h or for 72 h. We observed that the reduced percentage of differentiating neurons (Tuj^+^ or DCX^+^ neuroblasts/total number of cells) occurring in *Tis21*-null SVZ cultures, relative to wild-type, was reversed by silencing *Id3* expression or by treatment with BMP4 (**Figures 8B–E**).

In fact, the *Tis21*-null neurosphere cells targeted with shRNA to *Id3* showed a significantly increased percentage of early differentiated neuroblasts, relative to *Tis21*-null neurosphere cells targeted with shRNA to luciferase (TuJ^+^GFP^+^sh*Id3*/total GFP^+^sh*Id3* vs. TuJ^+^GFP^+^shLUC/total GFP^+^shLUC in *Tis21*-null neurosphere cells, *p* = 0.0008 after 48 h and *p* = 0.042 after 72 h; **Figure 8C**), as well as an increased percentage of the late differentiated neuroblasts (DCX^+^GFP^+^sh*Id3*/total GFP^+^sh*Id3* vs. DCX^+^GFP^+^shLUC/total GFP^+^shLUC in *Tis21*-null neurosphere cells, *p* = 0.022 after 48 h and *p* < 0.00001 after 72 h; **Figures 8B,C**). Similarly, the treatment with BMP4 significantly increased the percentage of either Tuj^+^ neuroblasts (TuJ^+^/total Hoechst^+^ cells in BMP4 treated vs. control treated *Tis21*-null neurosphere cells, *p* < 0.00001 after 48 h and after 72 h; **Figure 8E**) or of DCX^+^ neuroblasts, relative to control treated *Tis21*-null cells (DCX^+^/total Hoechst^+^ in BMP4 treated vs. control treated *Tis21*-null neurosphere cells, *p* < 0.00001 after 48 h and after 72 h; **Figures 8D,E**). Quantitatively, the increase of DCX^+^ neuroblasts in *Tis21*-null cultures 72 h after *Id3* silencing or after BMP4 treatment was about fourfold and threefold, respectively (well above the basal level of differentiation observed in wild-type cells); whereas the increase in wild-type DCX^+^ neuroblasts was about 70% and 50%, respectively. We conclude that both the silencing of *Id3* and the treatment by BMP4 were able to rescue the defect of differentiation caused by *Tis21* knockout.

### IMPAIRED OLFACTORY DETECTION IN *Tis21* KNOCKOUT MICE

**FIGURE 9:**
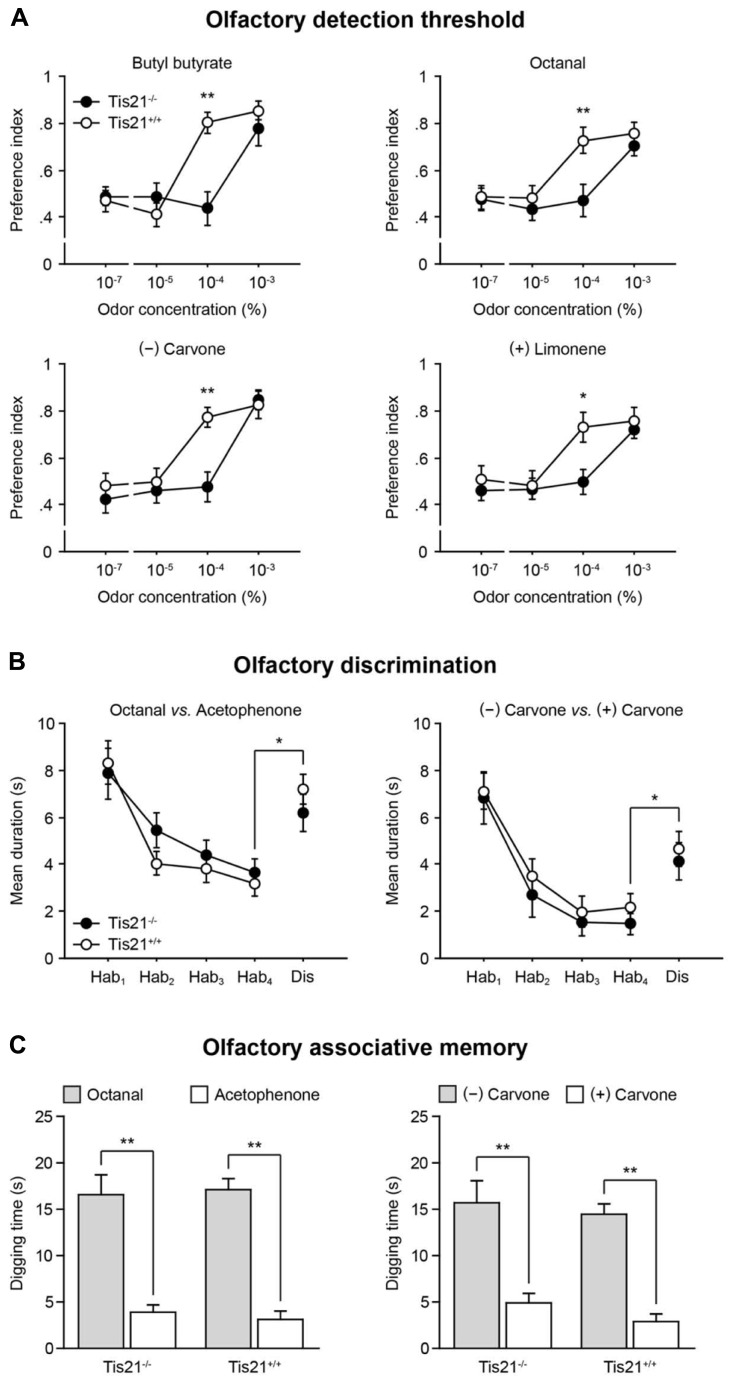
**Impaired olfactory detection in *Tis21* knockout mice. (A)** An olfactory detection threshold was determined by exposing the animals to series of progressively increasing concentrations of different odorants, in separate sessions, and measuring the time they spent investigating the odors vs. odorless mineral oil. Wild-type mice were able to detect the odors at a concentration equal to, or greater than, 10^-^^4^%, whereas *Tis21* knockout mice detected odors only at a concentration of 10^-^^3^%. **p < 0.01 and *p < 0.05 vs. wild-type. **(B)** The animals’ ability to discriminate between different or similar odors was analyzed by a habituation-dishabituation test, in which they were repeatedly exposed to one odor [octanal, or (-) carvone], during the habituation phase, and finally exposed to a different one [acetophenone, or (+) carvone] during the dishabituation trial. A significant increase of the sniffing time in the presence of the novel odor indicated normal discrimination, for both wild-type and *Tis21* knockout mice. Hab, habituation; Dis, dishabituation. *p < 0.05 vs. Hab-4. **(C)** Long-term, odor-reward, associative memory was evaluated by training for 4 days the animals to associate one odor [octanal, or (-) carvone] to a food reward, and then, on day five, the time they spent digging at the reinforced odor was compared to an unreinforced one [acetophenone, or (+) carvone]. In this test, both wild-type and *Tis21* knockout mice demonstrated a strong learning of the reinforced association. **p < 0.01 vs. unreinforced. Data are mean ± SEM.

Given the decrease of new SVZ-derived neurons observed in the *Tis21*-null OB, olfactory performances were assessed in wild-type and *Tis21* knockout mice, starting from their olfactory detection threshold. Animals were exposed to a series of progressively increasing concentrations of odorants [butyl butyrate, octanal, (-) carvone, and (+) limonene were used, in separate experimental sessions]; the time they spent investigating each of these odors vs. odorless mineral oil was measured (Breton-Provencher et al., 2009). An “odor preference” index was calculated, as the ratio between the time the animals spent investigating the odor and the total sniffing time, so that index values between 0.50 and 1.00 were indicative of preference for the odor. A significant increase in the time spent investigating the odor, compared to mineral oil, was considered as an indication of the animal’s odor detection capacity. For all the odorants, wild-type mice (*n* = 8) were able to detect the odor at a concentration equal to, or greater than, 10^-^^4^ %. Conversely, *Tis21* knockout mice (*n* = 10) were able to detect the odor only at a concentration of 10^-^^3^ %, which represents a significant increase of the olfactory detection threshold (**Figure 9A**; trial × genotype interaction: *F*[3,54] > 2.91, *p* < 0.04; two-way repeated measures ANOVA, followed by Fisher’s PLSD *post hoc* comparisons).

The animals’ ability to discriminate between odors was also assessed by a habituation-dishabituation test, which consisted of four habituation trials, in which the animals were repeatedly exposed to octanal, or (-) carvone, followed by one dishabituation trial, in which they were exposed to acetophenone, or (+) carvone (Gheusi et al., 2000). As a normal response, the time both wild-type (*n* = 6) and *Tis21* knockout (*n* = 8) mice spent investigating the odor during the habituation phase declined gradually. Conversely, it was significantly increased during the dishabituation phase (in the presence of the novel odorant), compared to the last habituation trial, with no statistically significant differences observed between groups (**Figure 9B**; effect of genotype: *F*[1,14] < 1.34, *p* > 0.27; effect of trial: *F*[1,14] > 48.73, *p* < 0.001; trial × genotype interaction: *F*[1,14] < 0.23, *p* > 0.63; two-way repeated measures ANOVA, followed by Fisher’s PLSD *post hoc* comparisons).

The animals’ long-term associative memory was finally assessed by a test based on rodents’ natural tendency to use olfactory cues to forage for food (Schellinck et al., 2001). Wild-type (*n* = 8) and *Tis21* knockout (*n* = 10) mice were trained for four consecutive days to associate octanal, or (-) carvone, with a sugar reward hidden beneath the surface of the wood chip bedding; then, on day five, the memory for the odor-reward association was evaluated by measuring the time the animals spent digging at such previously reinforced odors, compared to the time they spent digging at acetophenone, or (+) carvone, which were never reinforced during the training. In this test, with both odor pairs, wild-type and *Tis21* knockout mice demonstrated an equally strong learning of the reinforced association, by digging at reinforced odors significantly longer than they did at non-reinforced ones (**Figure 9C**; effect of genotype: *F*[1,18] < 0.95, *p* > 0.34; effect of odor: *F*[1,18] > 17.68, *p* < 0.001; odor × genotype interaction: *F*[4,72] < 0.98, *p* > 0.42; two-way repeated measures ANOVA, followed by Fisher’s PLSD *post hoc* comparisons).

Altogether, these data indicate that the altered olfactory bulb neurogenesis found in *Tis21* knockout mice selectively affected the animals’ ability to detect odors, sparing their capacity to discriminate between odors (either structurally different, or similar) and to retain long-term odor-reward associative memories.

## DISCUSSION

### *Tis21* REGULATES *BMP*, *Hes1/5* AND CELL CYCLE

*Tis21* appears to play a regulatory role in the SVZ at the intersection of the BMP and *Notch* pathways. In fact ablation of *Tis21* leads to strong inhibition of *BMP4* in the SVZ and in neurospheres, indicating for the first time that *BMP4* is activated *in vivo* by *Tis21*. Consistently, *Smad1* and *Smad8*, which are activated by *BMP4* and bind *Tis21* (Park et al., 2004; Miyazono et al., 2005), are reduced in *Tis21*-null neurospheres.

In addition, we obtain the first evidence that *Tis21* regulates *Id3*, *Id1*, and *Id2* in the SVZ, as ablation of *Tis21* strongly induces their expression in *Tis21*-null SVZ and neurospheres. We have previously shown that *Tis21* binds to the promoter and inhibits the transcription of *Id3* in the dentate gyrus (Farioli-Vecchioli et al., 2009). Id proteins, which have a HLH dimerization domain that lacks the DNA-binding domain, sequester E proteins, thus preventing their association to proneural bHLH transcription factors, in this way inactivating them (Lyden et al., 1999; Andres-Barquin et al., 2000; Yokota, 2001). One of these is *NeuroD1*, which is required for the differentiation of SVZ neurons (Gao et al., 2009) as well as for the maturation of hippocampal granule progenitor cells in differentiated neurons (Schwab et al., 2000). Consistently, *NeuroD1* expression is reduced in *Tis21*-null mice SVZ, compared to wild-type mice. Thus, it is very likely that the evident increase of *Id3*, *Id2*, *Id1* and decrease of *NeuroD1* expression play a role in the reduced differentiation of A neuroblasts observed in *Tis21*-null SVZ.

Given that the promoter of the anti-differentiative *Id3* gene is directly activated by *BMP4* through the Smad proteins (Miyazawa et al., 2002; Shepherd et al., 2008) and given that both these decrease in *Tis21*-null SVZ, this suggests that the direct inhibitory control by *Tis21* on *Id3* prevails on that by BMPs. Thus, *Tis21* may regulate the process of SVZ neurogenesis at least at two levels, by inducing *BMP4* and by inhibiting Id proteins. The induction of *BMP4* may prevent the amplification/proliferation of SVZ GFAP^+^ B stem cells, according to the antiproliferative action of *BMP4* on SVZ-derived neurospheres shown by Bonaguidi et al. (2005), and consistently with the proliferative effect of *Tis21* ablation that we observe in SVZ stem cells. Secondly, *Tis21*, by reducing the expression of the anti-differentiative Id proteins, may concomitantly favor the differentiation of neuroblasts. Such an opposite modulation exerted by *Tis21* on *BMP4* and *Id3* would also act as an upstream controller between the opposite actions on SVZ neural cells that these molecules, although in the same pathway, appear to have, i.e., antiproliferative for *BMP4* and proliferative as well as anti-differentiative for *Id3*. Notably, our data show that the differentiation defect of *Tis21*-null SVZ neurospheres is reverted not only by *Id3* silencing, but also by BMP4 treatment, suggesting that both molecules are part of the *Tis21* differentiative pathway in the SVZ. In fact, the increase of the number of differentiating neuroblasts by *BMP4* that we observed in both wild-type and *Tis21*-null neurospheres is consistent with the observations of Colak et al. (2008), showing that the number of DCX^+^ neuroblasts is positively controlled by the *BMP4*-*Smad* pathway. We can speculate that this pro-differentiative action of *BMP4* may either be intrinsic, or be simply a consequence of the antiproliferative action of *BMP4* on SVZ GFAP^+^ B stem cells and/or neurospheres.

As for the *Notch* pathway, we observed that *Hes5* and *Hes1* increase in *Tis21*-null SVZ and neurospheres. It is known that the role of *Notch1* in SVZ is to maintain stem cells (Ables et al., 2010; Aguirre et al., 2010), in particular those that are quiescent (Basak et al., 2012) and that the knockout of its mediator RBPJ induces proliferation and depletion of the stem cells pool (Imayoshi et al., 2010). *Hes1/5*, anti-neural effectors of *Notch1*, are coexpressed in the SVZ with *Notch1* (Stump et al., 2002); also, in the triple knockout of *Hes1/5/3* genes all neural stem cells differentiated prematurely into neurons and were depleted in most regions of the CNS including SVZ (Hatakeyama et al., 2004). Moreover, it is known that Id1-3 proteins inhibit neural stem cells from precocious neurogenesis also by directly inducing *Hes1* (Bai et al., 2007). Thus, the increased expression in *Tis21*-null SVZ of *Hes1* mRNA, and possibly of *Hes5*, may be explained with the loss of the direct inhibition of *Tis21* on *Id3*.

Furthermore, *Tis21* inhibits the S-phase of the cell cycle, acting directly at the promoter of *cyclin D1* (Farioli-Vecchioli et al., 2007); as *cyclin D1* activates the transcription of *Notch1* by recruiting CBP to its promoter (Kageyama et al., 2009; Latasa et al., 2009; Bienvenu et al., 2010), it is possible that *Tis21*, through its known direct negative control of *Id3* and *cyclin D1* (Farioli-Vecchioli et al., 2007, 2009), may also negatively regulate, though indirectly, the *Notch/Hes* pathway.

### CONTROL BY *Tis21* OF SVZ STEM/PROGENITOR CELLS QUIESCENCE AND TERMINAL DIFFERENTIATION

As observed here, *Tis21* appears to be required to maintain the quiescence of both active and slow-dividing B GFAP^+^ stem cells, identified by 2 h and by 14 day BrdU pulses, respectively, since their proliferation increases significantly in mice lacking *Tis21*. This result is consistent with the action of *Tis21* at more than one level, as an inhibitor of cell cycle molecules and Id proteins, and as an inducer of *BMP4* and of the *Hes1/5* genes. This action differs from that of *Notch1*, responsible for the maintenance primarily of quiescent rather than dividing stem cells (Basak et al., 2012), suggesting that *Tis21* controls stem cells amplification not only by Hes proteins but also, as mentioned above, through different pathways, such as BMP and the cell cycle.

**FIGURE 10:**
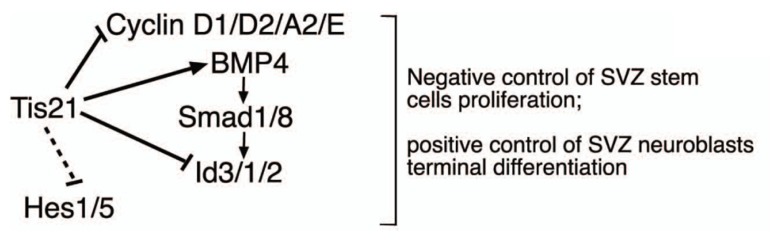
**Working model for the control of the adult SVZ neurogenesis by *Tis21*.** This model depicts in the SVZ the effectors by which *Tis21* inhibits proliferation of stem cells and is required for the differentiation of neuroblasts *in vivo* and *in vitro*. The silencing of *Id3* or the treatment with BMP4 in *Tis21*-null neurospheres rescues their defect of differentiation, indicating that by regulating the expression of these molecules *Tis21* acts as upstream controller of the opposite actions they exert in the SVZ, i.e., anti-differentiative for *Id3* and prodifferentiative for *BMP4*. The dotted lines indicate only correlative evidence. The negative regulation of *cyclin D1* by *Tis21* has been demonstrated in several neural and non-neural systems (Farioli-Vecchioli et al., 2007; Tirone et al., 2013), while the direct regulation of the *Id3* promoter by *Tis21* has been previously shown in neural cells (Farioli-Vecchioli et al., 2009).

However, the increased proliferation and amplification of stem cells in *Tis21*-null SVZ was associated with decreased generation of the dividing neuroblasts, without any detectable increase of apoptosis, while slow-dividing, post-mitotic neuroblasts increased (detected by 2 h and by 14 day BrdU pulses, respectively). Indeed, the decrease of dividing neuroblasts may be readily accounted for by the increased rate of proliferation of stem cells; in fact, when the neural stem/progenitor cells overproliferate, e.g., in consequence of an increase of *cyclin D1*, as occurs here, this leads to an acceleration of the cell cycle and, in the short period, to their decreased exit from the cell cycle and differentiation (see Artegiani et al., 2011). Consistently, an acceleration of the cell cycle has also been observed in the *Tis21*-null neural progenitors of the dentate gyrus (Farioli-Vecchioli et al., 2009). In contrast, the slow-dividing neuroblasts are cells that have exited the cell cycle and should have completed their terminal differentiation and migration to the olfactory bulb, rather than accumulate in the SVZ. Thus, although the increase of slow-dividing neuroblasts within the SVZ may be accounted for by the increased generation of stem/progenitor cells that in the long period have differentiated, this accumulation also suggests that *Tis21*-null neuroblasts present a defect in terminal differentiation and/or migration. Consistently, we observe a significant decrease in the number of neurons migrating throughout the RMS and in the olfactory bulb. However, direct analysis of the migration of SVZ neurons, performed in SVZ explants, indicates that there is no difference in the intrinsic ability to migrate of neural stem cells and neurons. Consequently, it appears that *Tis21*-null neuroblasts present a defect of terminal differentiation, rather than of migration; molecularly, it may depend on the increase of Id proteins as well as on the decrease of *BMP4* expression, as we show in *Tis21*-null neurosphere-derived neuroblasts that either silencing *Id3* or treating them with exogenous BMP4 in both cases restores their defect of differentiation. A working model of the role of *Tis21* in the SVZ adult neurogenesis and its interaction with the *BMP4*, *Ids*, and *Hes* gene pathways is depicted in **Figure 10**.

Thus, *Tis21* appears to control two main processes in the SVZ: the proliferation of stem cells and the terminal differentiation of neuroblasts and, indirectly, their migration to the OB. It is worth noting, however, that in the cerebellum the migration of cerebellar GCs is directly controlled by *Tis21* through different genes, such as the chemokine Cxcl3, the ephrin Efna4, or the regulator of actin filaments Jmy, as we have previously shown (Farioli-Vecchioli et al., 2012b); but of these genes, only Jmy is expressed in SVZ neurospheres.

### DEFECTIVE OLFACTORY BEHAVIOR IN *Tis21* KNOCKOUT MICE

Extensive evidence points to a functional role in the olfactory bulb of SVZ adult neurogenesis and links to olfactory-associated behaviors the ongoing addition of new granule interneurons to the existing circuitry. Despite an increasing number of investigations, however, no definitive indication has been obtained about which mechanism, in the framework of the processing of olfactory stimuli, specifically profits from such a phenomenon. The excitation produced by a mitral cell on a granule interneuron through dendrodendritic contacts can cause inhibition of the neighboring mitral cells; this has suggested that adult-generated GCs may participate in the spatial decorrelation of neural representations of odors – that is, in minimizing the likelihood that those representations use the same neurons, or patterns of neural activity (Lazarini and Lledo, 2011; Gheusi et al., 2013; Lepousez et al., 2013, for comprehensive reviews). As a consequence, the behavioral discrimination between odors should be impaired by experimental manipulations aimed at inhibiting adult neurogenesis in the olfactory bulb. Unfortunately, controversial evidence for this hypothesis are present in the literature, which may depend on the difficulty of the discrimination tasks administered to the animals (Enwere et al., 2004; Imayoshi et al., 2008; Lazarini et al., 2009), as well as on the period (gestational period vs. adulthood) when adult neurogenesis is manipulated (Gheusi et al., 2000; Enwere et al., 2004; Bath et al., 2008; Imayoshi et al., 2008; Breton-Provencher et al., 2009; Lazarini et al., 2009). Albeit controversially, olfactory memory (both odor memory and odor-reward associative memory) has also been indicated as a function to which adult neurogenesis in the olfactory bulb could be related. Results reported in the literature range from complete absence of deficits (Imayoshi et al., 2008), until selective impairments of short-term olfactory memory (Breton-Provencher et al., 2009), or impairments in the long-term retention of learned odor-reward associations, with either positive or negative valence (Lazarini et al., 2009; Valley et al., 2009).

Our results show no impairment in both olfactory discrimination and odor-reward associative memory after genetic ablation of *Tis21* gene in the mouse, when tested with perceptually discrete odors. The possibility remains, however, that subtle deficits in olfactory discrimination could be detected, in *Tis21*-null mice, if perceptually similar odors were used in an identical experimental setting. Conversely, *Tis21*-null mice show a significant increase in the odor detection threshold, compared to control littermates, and this finding is robustly supported by repeated testing with different odorants. This result is in accordance with that reported in a previous work (Breton-Provencher et al., 2009), in which adult neurogenesis in the olfactory bulb was reduced by AraC infusion in the lateral ventricle of adult mice. As argued by the authors, the raising of the detection threshold can be charged to the reduction in the number of periglomerular cells (Wachowiak and Shipley, 2006), an explanation that may be reasonably valid for *Tis21*-null mice. It is worth noting that the observed increase of odor threshold that we observe after robust decrease of periglomerular cells (35%) could also be interpreted as a functional implication of SVZ-derived new neurons primarily in olfactory sensitivity, rather than in olfactory memory.

## Conflict of Interest Statement

The authors declare that the research was conducted in the absence of any commercial or financial relationships that could be construed as a potential conflict of interest.
